# Increasing carotenoid production in *Xanthophyllomyces dendrorhous/Phaffia rhodozyma*: SREBP pathway activation and promoter engineering

**DOI:** 10.1186/s40659-024-00559-1

**Published:** 2024-11-05

**Authors:** Alejandro Durán, Maximiliano Venegas, Salvador Barahona, Dionisia Sepúlveda, Marcelo Baeza, Víctor Cifuentes, Jennifer Alcaíno

**Affiliations:** 1 Departamento de Ciencias Ecológicas, Facultad de Ciencias, Universidad de Chile, Las Palmeras 3425, Santiago, Chile; 2 Facultad de Ciencias, Universidad de Chile, Las Palmeras 3425, Santiago, Chile

**Keywords:** Astaxanthin, Gene expression regulation, Metabolic engineering, Mevalonate pathway, SREBP/Sre1 transcription factor, Promoter replacement

## Abstract

The yeast *Xanthophyllomyces dendrorhous* synthesizes astaxanthin, a high-value carotenoid with biotechnological relevance in the nutraceutical and aquaculture industries. However, enhancing carotenoid production through strain engineering remains an ongoing challenge. Recent studies have demonstrated that carotenogenesis in *X. dendrorhous* is regulated by the SREBP pathway, which includes the transcription factor Sre1, particularly in the mevalonate pathway that also produces precursors used for ergosterol synthesis. In this study, we explored a novel approach to enhance carotenoid synthesis by replacing the native *crtE* promoter, which drives geranylgeranyl pyrophosphate synthesis (the step where carotenogenesis diverges from ergosterol biosynthesis), with the promoter of the *HMGS* gene, which encodes 3-hydroxy-3-methylglutaryl-CoA synthase from the mevalonate pathway. The impact of this substitution was evaluated in two mutant strains that already overproduce carotenoids due to the presence of an active Sre1 transcription factor: *CBS.cyp61-*, which does not produce ergosterol and strain *CBS.SRE1N.FLAG*, which constitutively expresses the active form of Sre1. Wild-type strain CBS6938 was used as a control. Our results showed that this modification increased the *crtE* transcript levels more than threefold and fourfold in CBS*.cyp61*^*−*^*.pHMGS/crtE* and CBS*.SRE1N*.*FLAG*.*pHMGS/crtE*, respectively, resulting in 1.43-fold and 1.22-fold increases in carotenoid production. In contrast, this modification did not produce significant changes in the wild-type strain, which lacks the active Sre1 transcription factor under the same culture conditions. This study highlights the potential of promoter substitution strategies involving genes regulated by Sre1 to enhance carotenoid production, specifically in strains where the SREBP pathway is activated, offering a promising avenue for strain improvement in industrial applications.

**Supplementary Information:**

The online version contains supplementary material available at 10.1186/s40659-024-00559-1.

## Background

Carotenoids are natural yellow, orange and red liposoluble pigments produced by plants and algae, as well as some bacteria, archaea, and fungi. Among carotenoids, astaxanthin stands out due to its exceptional antioxidant properties and diverse applications in industries such as cosmetics, pharmaceuticals, and aquaculture [1, 2]. The basidiomycetous yeast *Xanthophyllomyces dendrorhous* (*Phaffia rhodozyma*) is one of the few organisms that naturally produces this pigment [3], making it a promising source for the biotechnological production of astaxanthin. Despite promising efforts to increase astaxanthin production in *X. dendrorhous*, such as strain improvements and optimization of culture conditions [4–7], enhancing carotenoid production through strain engineering remains challenging due to the complex regulatory networks that govern carotenoid biosynthesis.

**Fig. 1:**
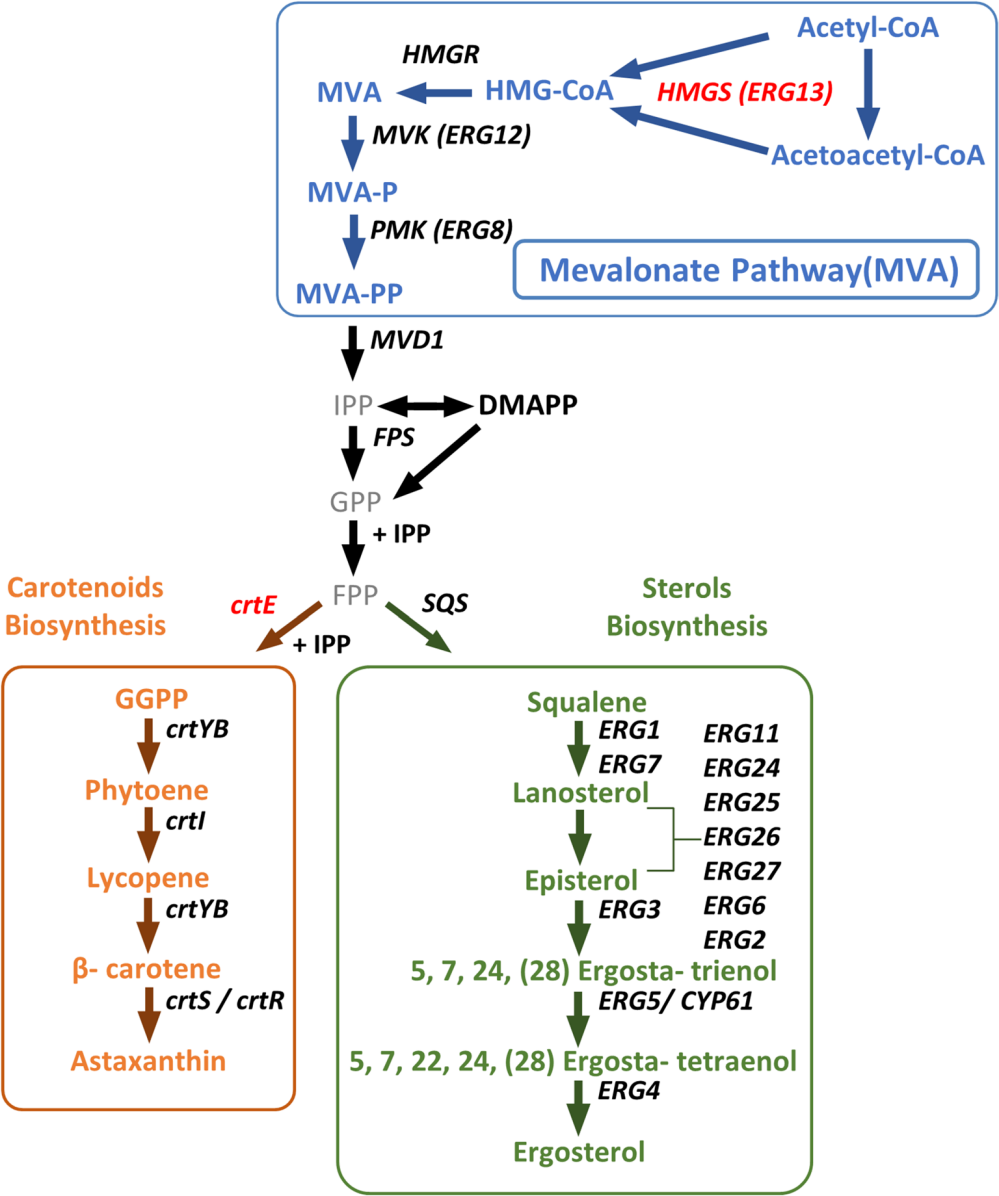
Mevalonate pathway, astaxanthin and ergosterol biosynthesis. Pathways are delineated by boxes of different colors: carotenoid biosynthesis (orange), sterol biosynthesis (green); and the MVA pathway (blue). Metabolites abbreviations: HMG-CoA (3-hydroxy-3-methylglutaryl-CoA), MVA (mevalonate), MVA-P (mevalonate-5-phosphate), MVA-PP (mevalonate-5-pyrophosphate), IPP (isopentenyl pyrophosphate), DMAPPP (dimethylallyl pyrophosphate), GPP (geranyl pyrophosphate), FPP (farnesyl pyrophosphate), and GGPP (geranylgeranyl pyrophosphate). Genes encoding the enzymes that catalyze each step are in italics, and the codes for those described in *X. dendrorhous* are as follows: *ERG10* (KX267759), *HMGS*/*ERG13* (MK368600/XDEN_03265), *HMGR* (AJ884949), IDI (DQ235686), FPS (KJ140284). Genes that control the astaxanthin and ergosterol synthesis pathway: *crtE* (DQ012943), *crtYB* (DQ016503), *crtI* (Y15007), *crtS* (EU713462), *crtR* (EU884133), *ERG11*/*CYP51* (KP317478), *ERG6* (LN48333.2), *ERG3* (MN930922) and *ERG5*/*CYP61* (JX183240), *ERG4* (MN930923) (Figure modified from Loto et al. [24])

Carotenoid synthesis in *X. dendrorhous* begins with the condensation of two acetyl-CoA molecules and shares common steps with the ergosterol pathway, including the mevalonate pathway (MVA) and the formation of farnesyl pyrophosphate (FPP) (Fig. 1). The conversion of FPP to geranylgeranyl pyrophosphate (GGPP), catalyzed by GGPP synthase encoded by the *crtE* gene, is the first committed step in carotenoid biosynthesis [8, 9]. Both carotenoid and sterol biosynthesis in *X. dendrorhous* are regulated by the Sterol Regulatory Element-Binding Protein (SREBP) pathway, a conserved lipid-sensing pathway involved in the maintenance of lipid homeostasis and metabolism in various organisms, which has been mostly studied in mammalian cells [10]. In this pathway, the SREBP transcription factor is initially synthesized as an inactive precursor anchored to the Endoplasmic Reticulum (ER) membrane through two transmembrane regions. Within the ER, the C-terminal domain of SREBP interacts with the protein SCAP (SREBP Cleavage-Activating Protein), which senses cellular sterol levels [11–13]. When cellular sterol levels are sufficient, SCAP retains SREBP within the ER by interacting with the protein INSIG (Insulin-Induced Gene). However, when sterol levels decline, the SCAP-SREBP complex is transported to the Golgi apparatus, where SREBP undergoes two successive proteolytic cleavages. The first is carried out by site-1 protease (S1P, a subtilisin-related serine protease) at the luminal loop, followed by site-2 protease (S2P, a metallopeptidase) within the first transmembrane segment of SREBP [14]. These cleavages release the N-terminal domain of SREBP, which then translocates to the nucleus to regulate the transcription of target genes by binding to Sterol Regulatory Elements (SREs) in their promoter regions [15, 16]. In yeasts such as *Schizosaccharomyces pombe* and *Cryptococcus neoformans*, homologs of SREBP (Sre1) and SCAP (Scp1) function similarly, regulating lipid metabolism based on sterol availability [17–19].

Recent studies have revealed the presence of an operational SREBP pathway in *X. dendrorhous*, which, besides regulating ergosterol biosynthesis, plays a role regulating carotenogenesis [20, 21]. Two key components of this pathway, Sre1 (SREBP homolog) and Stp1 (S2P homolog), have been identified and characterized [22, 23]. Through ChIP-exo analysis of Sre1, several Sre1-regulated genes were identified, including genes of the MVA pathway (*ERG10*, *HMGS* and *HMGR*), sterol biosynthesis (*ERG6*, *ERG7*, *ERG25*, *CYP51*, *crtR*) and carotenogenesis (*crtE* and *crtR*) [20]. Furthermore, RNA-seq analysis revealed a decrease in the transcript levels of *crtS* (which encodes the astaxanthin synthase) in *sre1*^−^ mutants derived from strains with an activated Sre1 transcription factor, and small ChIP-exo peaks were observed in the promoter region of this gene [20], suggesting that *crtS* might be regulated by Sre1 at some extent. Among the genes regulated by Sre1, *HMGS* exhibits one of the most significant transcript-level changes between strains with an active Sre1 transcription factor and *sre1*^−^ mutants [20, 22]. Given this, we hypothesized that expressing *crtE* under the regulation of the *HMGS* promoter, a direct target of Sre1, could enhance carotenoid production in strains having an active Sre1 transcription factor.

One of the earliest pieces of evidence suggesting the potential regulation of carotenoid production through the SREBP pathway in *X. dendrohous* was the observation of a carotenoid overproduction phenotype when ergosterol production was blocked by disrupting the *CYP61* gene (Fig. 1) [24]. Additionally, the production of sterols other than ergosterol also increased in the *cyp61*^−^ mutants. The phenotype observed in the *cyp61*^−^ mutants depends on the SREBP pathway as mutations preventing the activation of Sre1 restored carotenoid and sterol levels to those observed in the wild-type strain [22, 23]. Similarly, when the *SRE1* gene was replaced by a gene version exclusively expressing the Sre1 amino-terminal domain (Sre1N), sterol production increased, and carotenoid production nearly doubled compared to the wild-type strain [22].

Numerous efforts have been undertaken to enhance astaxanthin production in *X. dendrorhous*, including the overexpression of genes involved in the MVA pathway and in carotenogenesis [7]. In addition, the overexpression of *crtE* in *X. dendrorhous* significantly increased total carotenoid production [9], underscoring the crucial role of this gene in carotenogenesis. Then, considering that (i) mutants expressing the active form of Sre1 (*cyp61*^−^ and Sre1N mutants) overproduce carotenoids, (ii) the overexpression of *crtE* leads to increased carotenoid production, and (iii) *HMGS* is a prominent Sre1 target, this study aimed to investigate the impact of expressing the *crtE* gene under the regulation of the *HMGS* gene promoter on carotenoid production in *X. dendrorhous* strains with an active Sre1 transcription factor. This approach offers an innovative strategy to improve carotenoid production in *X. dendrorhous* by utilizing the regulatory role of the SREBP pathway.

## Methods

### Strains, plasmids, primers, media, and enzymes

The plasmids and strains used in this study are detailed in Table 1. Among plasmids, pBlueScript SK- (Stratagene) was employed for molecular cloning (Supplementary Fig. 1).

**Table 1:** Strains and plasmids used and built in this work

	Genotype or relevant features	Source or reference
Strains		
* E. coli*		
DH5α	Used for molecular cloning and plasmid maintenance	[29]
* X. dendrorhous*		
CBS*6938*	ATCC 96594, wild-type strain	ATTC
CBS*.cyp61*^−^	Zeo^r^. Strain derived from strain CBS*6938* in which the *CYP61* gene locus was disrupted with a module that confers resistance to zeocin (*Sh ble*)	[24]
CBS.*SRE1N.FLAG*	Hyg^r^. Strain derived from strain CBS*6938* strain in which the *SRE1* locus was replaced with a gene version that only expresses the active form of Sre1 (Sre1N)	[22]
CBS.p*HMGS/crtE*	Hyg^r^. Strain derived from strain CBS*6938*, in which the *crtE* gene promoter was replaced with the *HMGS* gene promoter	This work
CBS.*cyp61*^*−*^*.*p*HMGS/crtE*	Zeo^r^/Hyg^r^. Strain derived from strain CBS.*cyp61*^*−*^, in which the *crtE* gene promoter was replaced with the *HMGS* gene promoter	This work
CBS.*SRE1N*.*FLAG.*p*HMGS/crtE*	Zeo^r^/Hyg^r^. Strain derived from strain CBS*.SRE1N*.FLAG, in which the *crtE* gene promoter was replaced with the *HMGS* gene promoter	This work
Plasmids		
pBluescript SK- (pBS)	ColE1 ori; AmpR; cloning vector with blue-white selection	Stratagene
pBS-p*HMGS/crtE*	Plasmid pBlueScript SK- containing the module with the *HMGS* gene promoter (p*HMGS*) and the *crtE* gene segment (crtE*) at the *Eco*RV site	This work
pBS-*Up*-p*HMGS/crtE*	Plasmid pBlueScript SK-, containing the module with the *crtE* gene promoter upstream region (*UP*), the *HMGS* gene promoter (p*HMGS*) and the *crtE* gene segment (crtE*) at the *Eco*RV site	[24]
pBS-*Up-hph*-p*HMGS/crtE*	Plasmid pBlueScript SK-, containing the module comprising the *crtE* gene promoter upstream region (UP), the Hygromycin B resistance cassette (*hph*), the *HMGS* gene promoter (p*HMGS*) and the *crtE* gene segment (crtE*) at the *Eco*RV site	This work
pMN-*hph*	Plasmid pBlueScript SK-, containing the hygromycin B resistance cassette (1,8 kb) used for *X. dendrorhous* transformant selection at the *Eco*RV site	[51]

*CBS* Centraalbureau voor Schimmelcultures, *Utrecht* Netherlands, *ATCC* American Type Culture Collection, *Amp*^*s*^ sensitive to the antibiotic ampicillin, *Hyg*^*r*^ resistant to the antibiotic hygromycin B, *Zeo*^*r*^ resistant to the antibiotic zeocin

For plasmid propagation, the *E. coli* strain DH5α (Table 1) was used and cultivated in Lysogeny Broth (LB) medium (1% tryptone, 0.5% yeast extract, 0.5% NaCl) with constant agitation at 37 °C for 12 to 14 h. To select transformant colonies, semi-solid LB medium (1.5% agar) supplemented with ampicillin (100 µg/ml) and 5-bromo-4-chloro-3-indolyl-β-D-galactopyranoside, Xgal (120 µg/ml) was employed. *X. dendrorhous* strains were cultured in YM medium (0.3% yeast extract, 0.3% malt extract, 0.5% bactopeptone) supplemented with 1% glucose, with constant agitation at 22 °C. For the selection of yeast transformant colonies, semi-solid YM medium (1.5% agar) supplemented with hygromycin B (35 µg/ml) and/or zeocin (50 µg/ml) was used. *Escherichia coli* and *X. dendrorhous* were transformed by electroporation under the following conditions: 25 µF, 200 Ω, and 2.5 kV for *E. coli*, and 125 mF, 600 Ω and 0.45 kV for *X. dendrorhous*, using a BioRad Gene Pulser XcellTM (BioRad, Hercules, CA, USA). Electrocompetent yeast cells were prepared as described by Adrio et al. in 1995 [25] and Kim et al. in 1998 [26], and subsequently transformed with 5 to 10 μg of DNA.

The primers used in the PCR reactions are listed in Supplementary Table 1 and were synthesized by Integrated DNA Technologies (Coralville, IA, USA). Enzymes used in this work included DNA ligase T4, restriction endonucleases, Maxima reverse transcriptase, *Pfu*UltraII Fusion HS DNA polymerase, *Taq* DNA polymerase, DNase I, RNase A, T4 polynucleotide kinase, and FastAP Thermosensitive alkaline phosphatase, which were purchased from Agilent Technologies (Santa Clara, CA, USA), ThermoScientific (Waltham, MA, USA), and Life Technologies (Carlsbad, CA, USA), and used according to the manufacturer's instructions. The GeneRuler 1 kb Plus DNA Ladder from Thermo Scientific (Waltham, MA, USA) was used as a molecular weight marker.

### DNA amplification by PCR, cDNA synthesis (RT), and real-time PCR (qPCR)

*Taq* DNA polymerase was used for PCR reactions to confirm plasmid construction and the integration of fragments into the *X. dendrorhous* genome. The *Pfu* DNA polymerase was employed to amplify DNA fragments used in constructing DNA modules, including the genomic version of the *crtE* gene and the *HMGS* gene promoter.

The PCR reactions were conducted in a final volume of 25 µl, including 1X PCR buffer (500 mM KCl, 200 mM Tris–HCl pH 8.4), 2 mM MgCl_2_, 0.2 µM of each dNTP (dATP, dTTP, dGTP, dCTP), 1 µM of each primer, 1U of DNA polymerase enzyme, and between 10 and 20 ng of template DNA. In the case of colony PCR, an *E. coli* colony was suspended in the PCR reaction as template DNA. Amplification was carried out using an Applied Biosystem 2720 thermocycler (Waltham, MA, USA) with the following program: initial denaturation at 94 °C for 3 min (5 min for colony PCR), 30 cycles of denaturation at 94 °C for 30 s, primer annealing at 55 °C for 30 s, and extension at 72 °C adjusted to the size of the amplicon (30 s for amplicons from cDNA ≤ 1 kb using *Pfu* DNA polymerase, and 3 min with *Taq* DNA polymerase). Following the cycles, a final extension step at 72 °C was conducted for 10 min, and subsequently, the reaction was kept at 4 °C until use.

RNA samples were treated with DNase I following the enzyme provider's instructions for cDNA synthesis. The RT reaction was performed using Maxima Reverse Transcriptase from Thermo Fisher Scientific (Waltham, MA, USA) in a final volume of 20 µl, with a specific volume of RNA (5 µg). To amplify the cDNA of each gene, a PCR reaction was conducted as previously indicated. For qPCR reactions, the PCR reaction mix was prepared in accordance with the specifications of the SsoAdvanced Universal SYBR Green Supermix Kit from BioRad (Hercules, CA, USA), which included 10 µl of the kit mix, 8 µl of sterile water, 1 µl of RT, and 1 µl of the forward and reverse primer mix (10 µM each). The samples were loaded into the BIO RAD C1000 touch thermal cycler CFX96 Real-Time System. To assess transcript levels, Ct (threshold cycle) values were normalized to the value obtained for the ACT gene (Genbank: X89898.1) of *X. dendrorhous* and expressed relative to control conditions using the 2-ΔΔCt method [27].

### DNA extraction and purification

Plasmid DNA purification was performed using the GeneJET Plasmid Miniprep Kit from Thermo Fisher Scientific (Waltham, MA, USA) following the manufacturer's instructions. Ligation reactions involving digested plasmid DNA and insert DNA (module) were performed using the DNA ligase T4 enzyme, also in accordance with the manufacturer's instructions. The silica bead method was used to extract DNA fragments from agarose gels, as described by Boyle and Lew in 1995 [28]. Genomic DNA from *X. dendrorhous* was extracted using 0.5 mm glass beads [29]. For yeast RNA extraction, 1 ml of a yeast culture was collected, and the extraction was carried out according to Chomczynski and Sacchi in 1987 [30]. Subsequently, the obtained samples were quantified using the QubitTM 4 fluorometer with the QubitTM RNA BR Assay Kit from Thermo Fisher Scientific (Waltham, MA, USA).

### Extraction of carotenoids and sterols, and RP-HPLC analysis

The extraction of carotenoids was performed using the acetone extraction method described by An et al. in 1989 [31]. Total carotenoids were quantified by measuring the absorbance at 474 nm, while sterols were quantified at 280 nm, and the values were normalized to the dry weight of the yeast. All analyses were conducted in triplicate. The carotenoid composition was determined using reverse-phase high-performance liquid chromatography (RP-HPLC) with an HPLC system equipped with a Shimadzu SPD-M10A diode array detector and an RP-18 LiChroCART^®^ 125–4 column (Merck KGaA, Darmstadt, Germany). The mobile phase used was acetonitrile: methanol: isopropanol in a 75:20:5 (v/v) ratio, and the flow rate was set at 1 ml/min under isocratic conditions. Carotenoids were identified based on their retention times and absorption spectra according to standards [32].

## Results

### Module construction to replace the *crtE* gene promoter with the *HMGS* gene promoter

To assess the impact of substituting the *crtE* gene promoter with the *HMGS* gene promoter in *X. dendrorhous* on carotenogenesis, a DNA module was constructed and inserted into the cloning vector pBluescript SK- (pBS) (Supplementary Fig. 1). Specific primers were designed to produce amplicons with complementary ends, enabling the fusion with adjacent fragments. This module was constructed by amplifying three distinct DNA fragments from the wild-type strain CBS*6938*: (i) a 512 bp fragment corresponding to the upstream region of the *crtE* gene promoter (*UP*), (ii) a 1,012 bp corresponding to the promoter region of the *HMGS* gene (*pHMGS*), and (iii) an 863 bp fragment encompassing the *crtE* gene from its translation start codon in exon 1 to exon 4 (crtE*). Through OE-PCR, the *pHMGS* and crtE* fragments were fused and subsequently inserted at the *Eco*RV site within the multiple cloning site of pBS. This process resulted in the generation of plasmid pBS-*pHMGS/crtE*. The forward primer designed for amplifying the *pHMGS* fragment included the recognition site for the *Hpa*I enzyme, which facilitated the integration of the *UP* fragment at this site, resulting the plasmid pBS-*UP*-*pHMGS/crtE*. To insert the hygromycin B resistance module (*hph*) between the *UP* and *pHMGS* fragments, the same procedure was employed. The reverse primer designed to amplify the *UP* fragment also contained an *Hpa*I site, enabling the integration of the hygromycin B resistance module, ultimately yielding the plasmid pBS-*UP*-*hph*-*pHMGS/crtE* (Supplementary Fig. 1). The obtained plasmids were confirmed by PCR, and the insert of pBS-*UP*-*hph-pHMGS/crtE* was sequenced to confirm that no mutations were introduced.

**Fig. 2:**
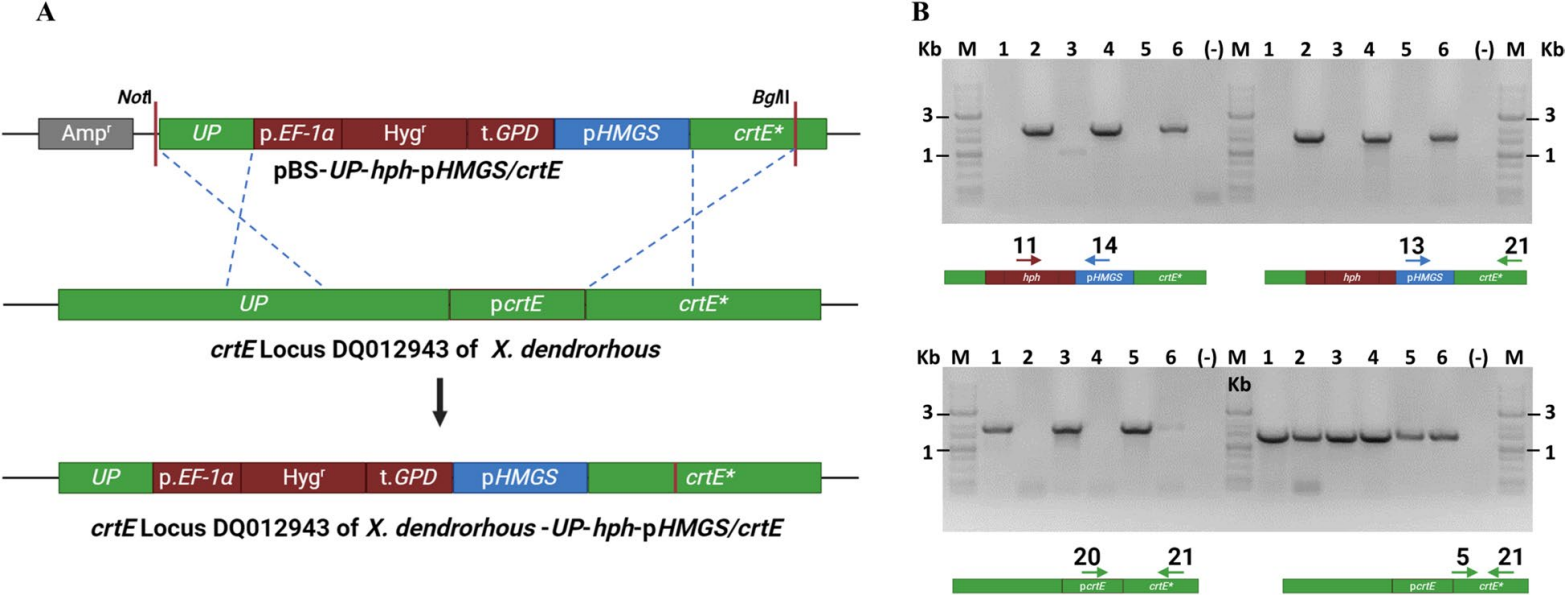
Representation of the integration of the *Up-hph-pHMGS*/*crtE* module in the *X. dendrorhous* genome. **A** Scheme illustrating the resulting product with the *Up-hph-pHMGS*/*crtE* module (digested with *Not*I and *Bgl*II enzymes), used to replace the native *crtE* gene promoter with the *HMGS* promoter through double homologous recombination. **B** PCR analysis of strains used in this work. A representation of the amplified fragment is provided beneath each gel, with primers indicated by numbered arrows according to Supplementary Table 1.2. Template DNA sources are as follows: CBS*6938* (lane 1), CBS.*pHMGS*/*crtE* (lane 2), CBS.*cyp61*^*−*^ (lane 3), CBS.*cyp61*^*−*^*.pHMGS*/*crtE* (lane 4), CBS.*SRE1N.FLAG* (lane 5), and CBS.*SRE1N.FLAG.pHMGS*/*crtE* (lane 6). The GeneRuler 1 kb Plus DNA Ladder (M) was used as a molecular weight marker, and (−) represents a negative control without DNA in the PCR reaction

The plasmid pBS-*UP*-*hph-pHMGS/crtE* was digested with *Not*I and *Bgl*II enzymes to release the transformant DNA module (Fig. 2A). This module was then used to transform the wild-type strain CBS*6938* and the mutants CBS*.cyp61*^*−*^ and CBS*.SRE1N*.*FLAG*, resulting in strains CBS*.pHMGS/crtE*, CBS*.cyp61*^*−*^*.pHMGS/crtE*, and CBS*.SRE1N*.*FLAG*.*pHMGS/crtE*, respectively. The successful replacement of the native promoter of the *crtE* gene with the *HMGS* gene promoter in the generated strains was confirmed through PCR analysis using appropriate primer pairs (Fig. 2B).

**Fig. 3:**
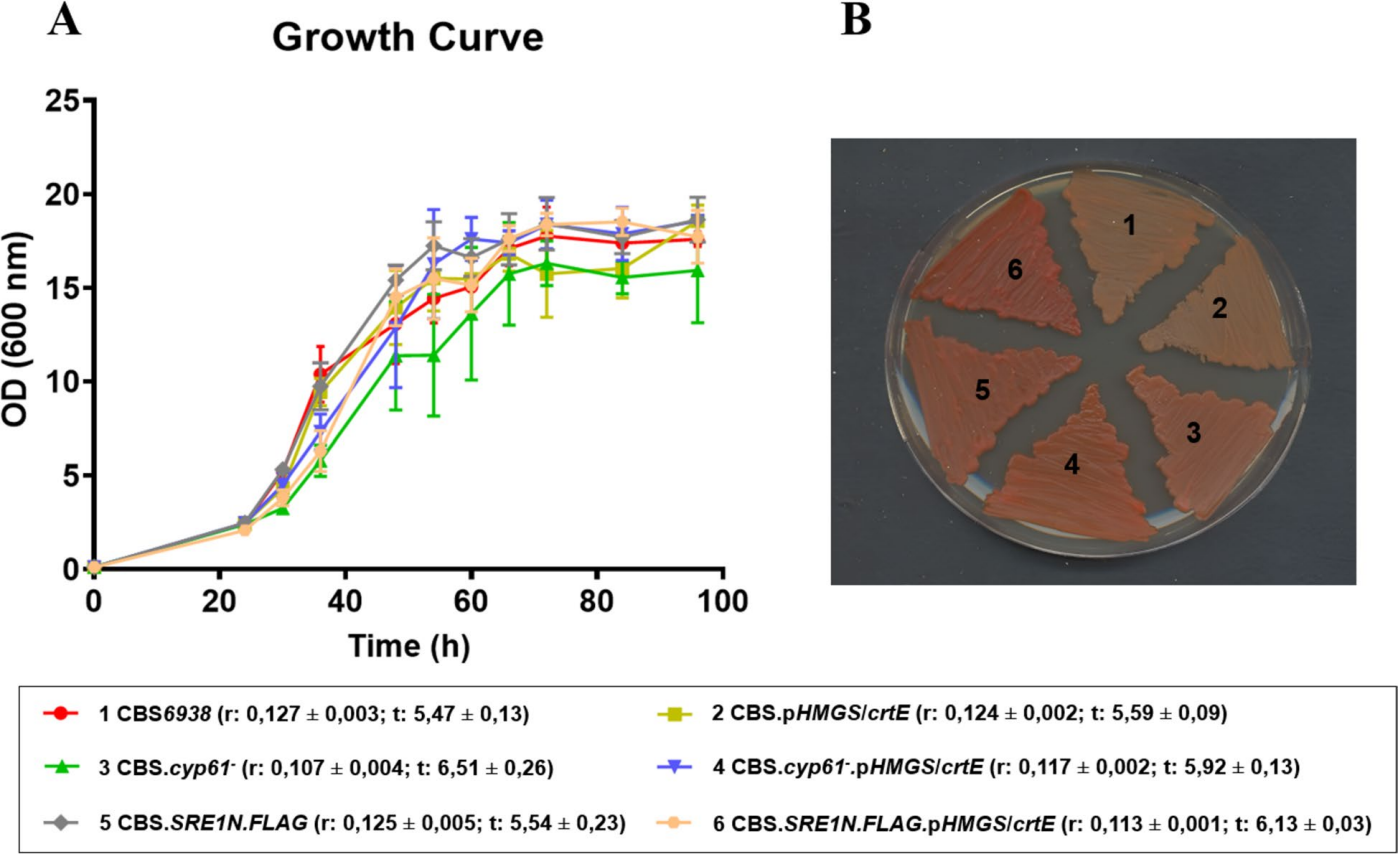
Growth curves and color phenotype of strains. **A** Growth curves of strains CBS*6938*, CBS.*cyp61*^*−*^, CBS.*SRE1N.FLAG* and their respective mutants: CBS.p*HMGS/crtE*, CBS.*cyp61*^*−*^*.*p*HMGS/crtE* and CBS.*SRE1N.FLAG.* p*HMGS/crtE*. The strains were cultured in liquid YM medium at 22 °C with constant agitation, and growth was monitored at 600 nm. Growth rate (r) and generation time (t) were determined based on the method described by Zwietering et al. in 1990 [50], and values represent the average ± standard deviation of three simultaneous cultures for each strain. It should be noted that growth parameters were estimated only as an approximation to assess significant alterations in growth. No statistically significant differences in growth parameters were observed between mutant and their respective parental strain (p-value < 0.01). **B** Strains cultured on YM agar plates (1.5%) at 22 °C for 72 h numbered as follows: CBS*6938* (1), CBS.*pHMGS/crtE* (2), CBS.*cyp61*^*−*^ (3), CBS.*cyp61*^*−*^*.SRE1.pHMGS/crtE* (4), CBS.*SRE1N.FLAG* (5), and CBS.*SRE1N.FLAG.pHMGS/crtE* (6)

To the naked eye, no noticeable color difference was observed between strain CBS*.pHMGS/crtE* and the wild-type. However, the same genetic modification in strains CBS*.cyp61*^*−*^ and CBS*.SRE1N*.*FLAG* resulted in strains displaying a more intense pigmentation than their respective parental strain (Fig. 3). These observations suggest that replacing the native promoter of the *crtE* gene with the *HMGS* gene promoter further enhances carotenogenesis in carotenoid-overproducing strains with an active SREBP pathway.

Subsequently, the three strains obtained in this work and their corresponding parental strains were cultured in triplicate in YM medium at 22 °C with constant agitation. Growth curves were constructed, and growth rates (r) and generation times (t) were estimated (Fig. 3). In general terms, the assessed genetic modification did not impact growth under the studied conditions, as all six strains exhibited similar growth curves and growth parameters (Fig. 3A). After 120 h of culture (stationary growth phase), samples were collected to extract RNA, carotenoids, and sterols.

### Transcript level of the *crtE* is enhanced when it is regulated by the *HMGS* gene promoter

**Fig. 4:**
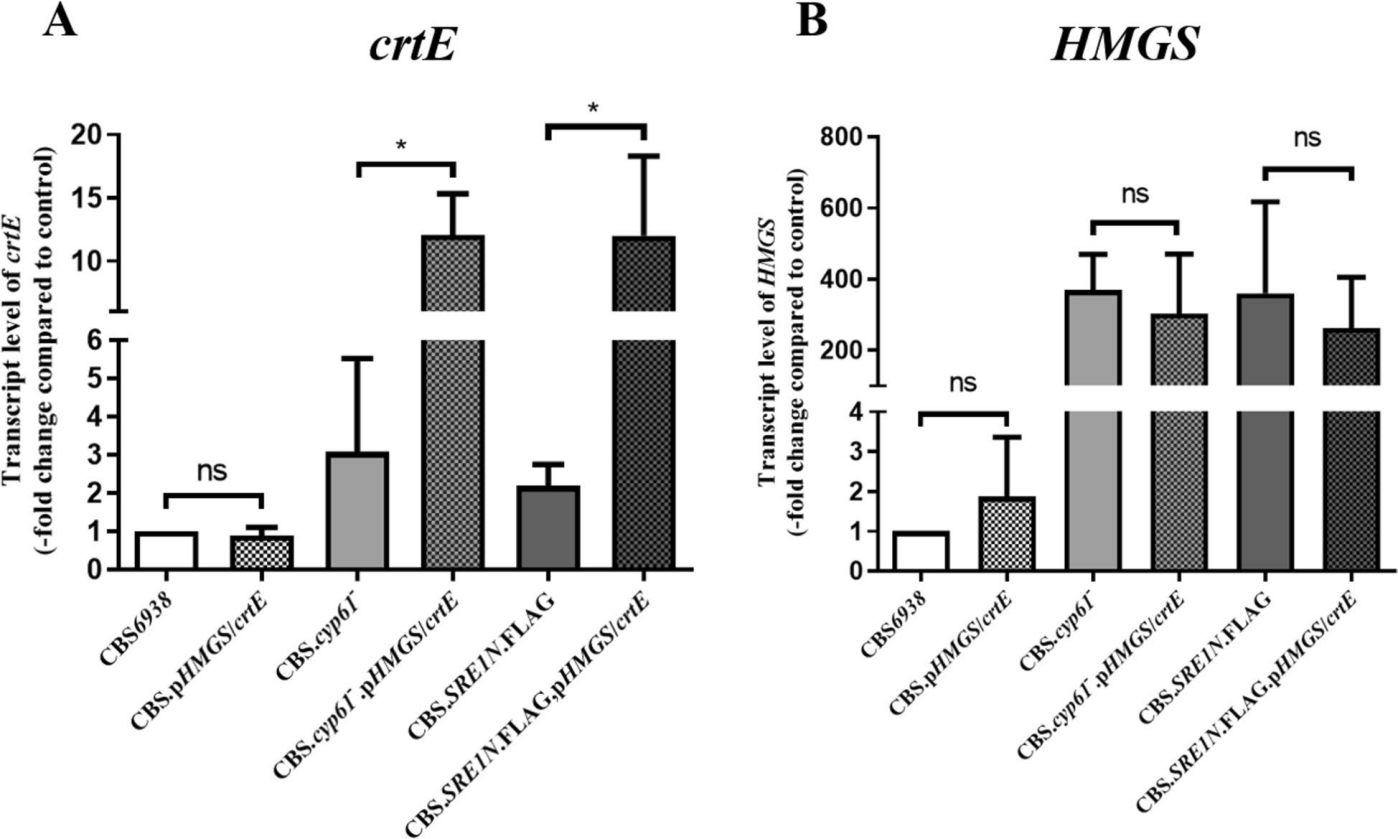
Transcript levels of the *crtE* and *HMGS* genes in *X. dendrorhous* strains in this work. Relative transcript levels of genes (**A**) *crtE* (GenBank: DQ012943) and (**B**) *HMGS* (GenBank: MK368600) were determined by RT-qPCR after 120 h of culture, normalized to the housekeeping gene encoding β-actin (GenBank: X89898.1), and then with respect to the wild-type strain CBS*6938*, which was set as 1. Values are the average ± standard deviation of three independent experiments. *ns* non-significant differences and *p-value < 0.05, one-way ANOVA test and student’s t-test)

To assess whether replacing the native promoter of the *crtE* gene with that of the *HMGS* gene effectively increases *crtE* transcript levels, RT-qPCR analysis was performed on the six strains studied in this work. Gene *HMGS* was included in this analysis to evaluate if the created modification affected its expression, at least at the transcript level. The relative expression of both genes was normalized to the expression of the actin gene. As expected, the expression of both genes was higher in strains CBS*.cyp61*^*−*^ and CBS*.SRE1N*.*FLAG* when compared to the wild-type, as these genes are Sre1 targets [20], and both strains should have an active Sre1N under the cultured conditions, as confirmed previously [23]. Interestingly, the RT-qPCR analysis revealed that the expression of *crtE* increased approximately three and four-fold in strains CBS*.cyp61*^*−*^*.pHMGS/crtE* and CBS*.SRE1N*.*FLAG*.*pHMGS/crtE* strains, respectively, when compared to their corresponding parental strain (*p-value < 0.05). However, the CBS*.pHMGS/crtE* strain did not show significant changes compared to the wild-type (Fig. 4A). Regarding the *HMGS* gene transcript levels, none of the generated strains in this work showed significant differences when compared to their corresponding parental strain (Fig. 4B). These results confirm that the expression of the *crtE* gene at the transcript level is enhanced when it is regulated by the *HMGS* gene promoter in strains with the active transcription factor Sre1N, without affecting the *HMGS* transcript levels.

### Carotenoid production increases in strains with an active Sre1 transcription factor when the *crtE* gene is regulated by the *HMGS* promoter

**Fig. 5:**
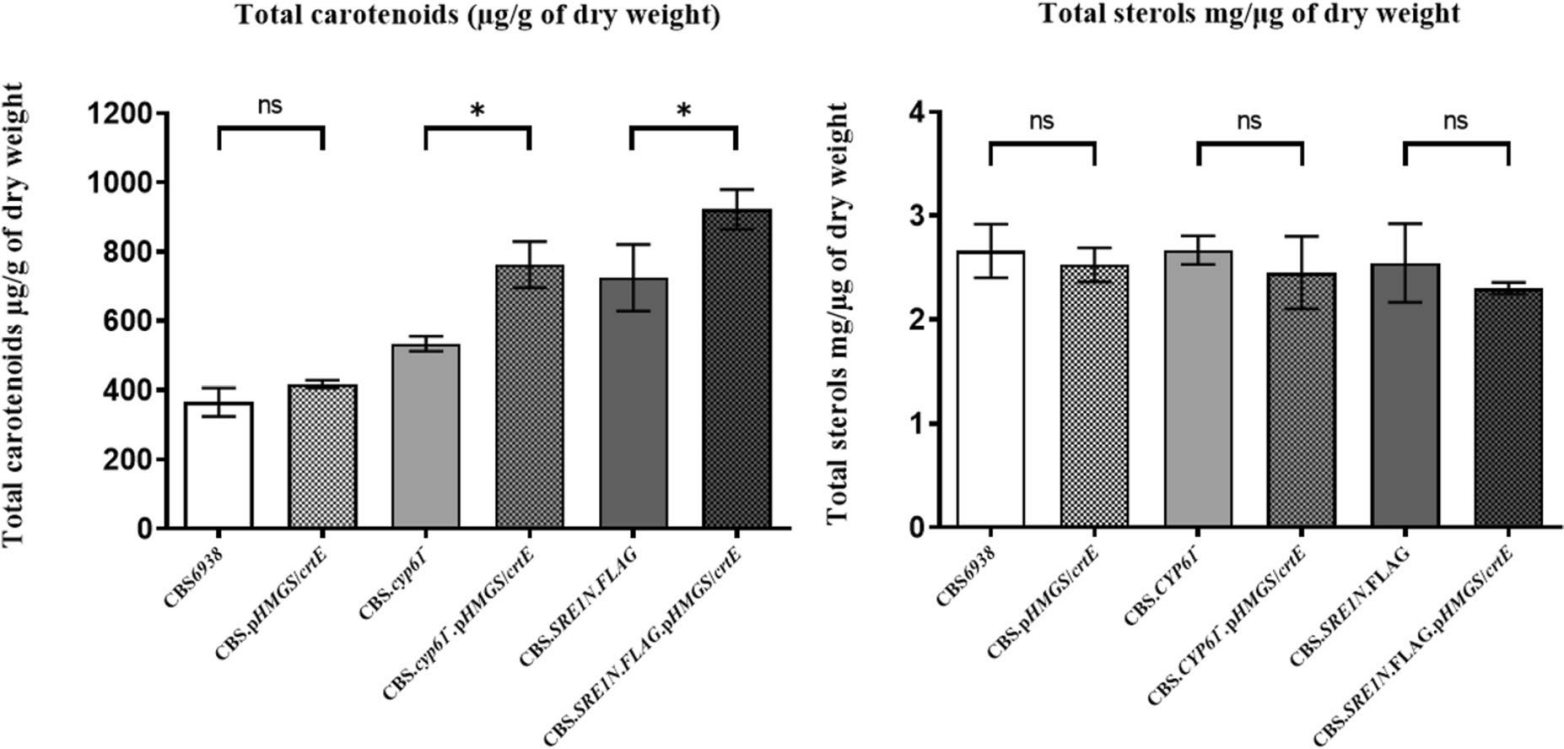
Carotenoids and sterols in *X. dendrorhous* strains studied in this work.** A** Carotenoids and **B** sterols were extracted at the stationary phase of growth cultures (120 h of culture) and quantified at 474 nm and 280 nm, respectively. Values correspond to the average value from three independent cultures of each strain, and the error bars correspond to the standard deviation. Data were normalized with respect to the dry weight of the yeast. *ns* non-significant differences and *p-value < 0.05, student’s t-test

After 120 h of cultivation of the six strains analyzed in this work, samples were taken to extract carotenoids and sterols, which were quantified spectrophotometrically, and carotenoid composition was analyzed by RP-HPLC. No significant differences in carotenoid amount and composition were observed between strains CBS*6938* and CBS*.pHMGS/crtE*. However, when comparing the parental strains CBS*.cyp61*^*−*^ and CBS*.SRE1N*.*FLAG* with their respective transformant strains, a significant increase in total carotenoid production was observed in CBS*.cyp61*^*−*^*.pHMGS/crtE* and CBS*.SRE1N*.*FLAG*.*pHMGS/crtE* strains, with an increase of 1.43 and 1.22 times, respectively (Fig. 5A). Regarding carotenoid composition, the CBS*.cyp61*^*−*^*.pHMGS/crtE* strain exhibited a two to three-fold increase in β-carotene, intermediate carotenoids between β-carotene and astaxanthin, and of other carotenoids, with a slight increment in astaxanthin content. Similarly, the mutant strain CBS*.SRE1N*.*FLAG*.*pHMGS/crtE* also displayed an elevated content of β-carotene, intermediates, and other carotenoids, but the amount of astaxanthin remained the same, resulting in a decreased proportion of astaxanthin relative to other carotenoids in this strain (Table 2).

**Table 2:** Composition of carotenoids in *X. dendrorhous* strains studied in this work

Carotenoid	Strains					
	CBS*6938*	CBS*.* p*HMGS*/*crtE*	CBS.*cyp61*^−^	CBS*.cyp61*^*-*^. p*HMGS*/*crtE*	CBS*.SRE1N*. FLAG	CBS*.SRE1N* FLAG p*HMGS*/*crtE*
Astaxanthin	320.0 ± 34.8 **(87.6 ± 3.3)**	339.0 ± 21.6 **(81.1 ± 3.6)**	440.1 ± 24.2 **(82.5 ± 2.9)**	572.7 ± 88.6* **(74.2 ± 7.6)**	659.8 ± 44.0 **(86.9 ± 1.8)**	652.0 ± 40.3 **(70.7 ± 1.5)**
Phoenicoxanthin	15.4 ± 2.9 **(4.2 ± 0.5)**	23.7 ± 2.3* **(5.7 ± 0.5)**	21.9 ± 7.6 **(4.1 ± 1.3)**	24.7 ± 8.4 **(3.2 ± 0.9)**	16.4 ± 1.9 **(2.2 ± 0.3)**	22.0 ± 2.5 **(2.4 ± 0.1)**
OH-equinenone	4.2 ± 2.6 **(1.1 ± 0.6)**	7.5 ± 4.0* **(1.8 ± 1.0)**	9.9 ± 2.4 **(1.9 ± 0.5)**	30.7 ± 7.8* **(4.0 ± 1.2)**	9.4 ± 2.8 **(1.2 ± 0.4)**	40.3 ± 9.2* **(4.3 ± 0.8)**
Equinenone	6.0 ± 2.3 **(1.6 ± 0.5)**	5.7 ± 1.3 **(1.4 ± 0.3)**	7.6 ± 0.9 **(1.4 ± 0.2)**	5.1 ± 0.6* **(0.7 ± 0.1)**	9.0 ± 0.5 **(1.2 ± 0.0)**	10.1 ± 3.7 **(1.1 ± 0.4)**
β-carotene	4.5 ± 1.9 **(1.2 ± 0.5)**	9.2 ± 4.2* **(2.2 ± 1.1)**	21.6 ± 6.5 **(4.1 ± 1.3)**	57.2 ± 27.3* **(7.5 ± 4.0)**	23.4 ± 1.6 **(3.1 ± 0.3)**	73.7 ± 1.6* **(8.0 ± 0.4)**
Phytoene	ND	ND	ND	ND	ND	ND
Other carotenoids	15.5 ± 8.4 **(4.2 ± 2.0)**	32.7 ± 15.1* **(7.8 ± 3.7)**	32.7 ± 11.3 **(6.1 ± 2.1)**	79.4 ± 11.3* **(10.4 ± 4.1)**	40.9 ± 12.1 **(5.4 ± 1.6)**	123.6 ± 13.4* **(13.4 ± 2.2)**
Total carotenoids	366 ± 41.1 **(100)**	417.8 ± 10.4 **(100)**	533.6 ± 21.8 **(100)**	769.9* ± 63.3 **(100)**	758.8 ± 37.1 **(100)**	921.8 ± 57.6 **(100)**

Carotenoids were extracted after 120 h of culture. Values are expressed in ppm (μg per g of dry yeast). Data in bold and in parentheses correspond to the percentage of each carotenoid relative to the total amount of carotenoids. The table shows the average ± standard deviation of three biological replicates. *ND* not detected. Other carotenoids include: torulene, OH-k-torulene, canthaxanthin, and OH-K-γ-carotene. Significant differences using a t-Student test (p-value < 0.05) between each mutant strain compared to its respective parental strain are indicated in the table (*)

Since the step catalyzed by the *crtE* gene product corresponds to the first specific step of carotenoid synthesis, where it diverges from sterol synthesis, an assessment of sterol production was conducted to evaluate the potential impact of increased carotenoid production on sterol levels. No significant differences were observed in sterol production between the mutant strains and their respective parental strains (Fig. 5B). Therefore, the *crtE* gene promoter replacement did not affect sterol production in these strains.

## Discussion

*X. dendrorhous* is a promising and efficient natural carotenoid and astaxanthin production source. Its fermentation process offers advantages due to its relatively short growth time [33], simple culture requirements [34], and the fact that it can use low-cost substrates, including food waste [35, 36], making this yeast an environmentally sustainable alternative that also enhances economic benefits. Moreover, this yeast can be efficiently genetically modified using recombinant DNA technology [6], facilitating studies of metabolic pathways and developing alternative modified strains. Redirecting flux towards carotenoid synthesis has proven to be an efficient strategy to improve production in *X. dendrorhous*. In this sense, inhibiting competing pathways like fatty acid and ergosterol synthesis while promoting flux towards the MVA pathway significantly increases astaxanthin production in *X. dendrorhous*. For example, the supplementation of penicillin and ethanol (promoters of the MVA pathway), combined with triclosan (an inhibitor of fatty acids synthesis) and fluconazole (an inhibitor of ergosterol biosynthesis), increased the astaxanthin yield by 51% in this yeast [34].

In this study, we employed a novel approach to enhance carotenoid production in *X. dendrorhous* by increasing *crtE* transcript levels, which encodes the enzyme involved in the synthesis of GGPP [9], the first specific step in carotenoid biosynthesis. Other works overexpressed the *crtE* gene in *X. dendrorhous* [37–39]; however, this work considered a novel strategy by replacing the *crtE* gene promoter with that of the *HMGS* gene, which contains SRE (Sterol Regulatory Element) sites. This approach allowed us to evaluate how this promoter change (p*HMGS/crtE*) impacts *crtE* expression and, consequently, carotenoid production in both wild-type and strains having the active Sre1 transcription factor.

The strains modified in this work derived from three distinct parental strains: CBS*6938*, which served as the wild-type strain; CBS*.cyp61*^*−*^, a mutant that does not produce ergosterol but exhibits an overproduction of carotenoids [24]; and CBS*.SRE1N*.*FLAG*, a mutant that exclusively expresses the active form of Sre1 that also overproduces carotenoids [22]. Previous western blot analysis confirmed that strains *CBS.cyp61*^*−*^ and CBS*.SRE1N*.*FLAG* have the active form of the transcription factor Sre1 (Sre1N), unlike the wild-type strain [23] under the same culture conditions used in this work. Both strains CBS*.cyp61*^*−*^*.pHMGS/crtE* and CBS*.SRE1N*.*FLAG*.*pHMGS/crtE* displayed noticeable phenotypic color changes characterized by increased intensity and a reddish hue, with a significantly higher carotenoid content than CBS*6938* and their respective parental strains. In contrast, the wild-type derived strain CBS*.pHMGS/crtE* did not show any visible alterations in pigmentation, suggesting that the promoter replacement only benefits carotenoid production in strains with an active Sre1N transcription factor. The levels of the *crtE* transcripts in the mutant strains CBS.*cyp61*^*−*^*.pHMGS*/*crtE* and CBS.*SRE1N.FLAG.pHMGS*/*crtE* increased up to four-fold compared to their respective parental strains. No such increase was observed in CBS*.pHMGS/crtE*, consistent with the absence (or basal levels) of Sre1N in the wild-type strain. These results align with previous findings that demonstrated the importance of SRE sites in the *HMGS* promoter for gene regulation via the SREBP pathway [22, 40].

Previous studies have reported that *cyp61*^−^ mutants produce higher amounts of carotenoids than wild-type strains [24, 41], while deletion of the *SRE1* gene in CBS*.cyp61*^*−*^ reduces carotenoid production [22]. This suggests that the inability to produce ergosterol and the accumulation of intermediate sterols in strain CBS*.cyp61*^*−*^ may trigger a sterol-dependent mechanism that promotes carotenoid and astaxanthin overproduction [24, 42]. Furthermore, it is well-established that the SREBP pathway is activated in both yeast and higher eukaryotes through a mechanism regulated by cellular sterol levels [17, 19, 22, 43]. Previously was reported that the CBS*.SRE1N*.*FLAG* strain, which expresses only the active form of Sre1, has a substantial increase in total carotenoid production (691 µg/g yeast dry weight) compared to CBS*6938* (302 µg/g yeast dry weight) and surpasses the carotenoid content of strain CBS*.cyp61*^*−*^ by 11.6% [22]. No significant changes in sterol production were observed between the mutant strains obtained in this work and their corresponding parental strains. Similarly, in another study where the *crtE* gene was overexpressed under the regulation of the glyceraldehyde-3-phosphate dehydrogenase gene promoter and integrated into the multiple copy ribosomal DNA sequence region of the *X. dendrohous* genome, a higher astaxanthin content was observed, and ergosterol production did not show major changes [8].

Although a significant increment of carotenoids was observed in strains harboring the active Sre1N transcription factor, astaxanthin levels did not increase as markedly. This may represent a limitation of the strategy used in this work. In particular, the mutant strain CBS*.cyp61*^*−*^*.pHMGS/crtE* showed a significantly different carotenoid composition than its parental strain, exhibiting increased astaxanthin levels, although its proportion relative to total carotenoids decreased. Enhanced astaxanthin production in *X. dendrorhous cyp61*^−^ mutants of about 1.4-fold was previously reported [41], and this genetic modification also resulted in the accumulation of intermediate carotenoids between β-carotene and astaxanthin [24]. These observations indicate that besides increasing carotenoid production, the disruption of the *CYP61* gene provides an opportunity for further modifications to increment astaxanthin levels due to the accumulation of intermediary carotenoids. A similar outcome was observed in strain CBS*.SRE1N*.*FLAG*.*pHMGS/crtE*, which showed no significant changes in astaxanthin quantity compared to its parental strain. These results indicate that the modification made to the *crtE* gene in this work promotes overall carotenoid production in strains with an active Sre1N transcription factor (CBS*.cyp61*^*−*^*.pHMGS/crtE* and CBS*.SRE1N*.*FLAG*.*pHMGS/crtE*) and opens up an opportunity for further enhancement of astaxanthin production in these strains.

Other works have reported that overexpressing the *crtE* gene successfully increases carotenoid production in *X. dendrorhous*. For example, in a study that compared carotenoid production between a wild-type strain and various mutants deriving from it, the introduction of an additional copy of the *crtE* gene resulted in an increment of about 36% of total carotenoids (from 376.3 to 513.4 µg/g yeast dry weight) compared to the parental strain after 72 h of cultivation [9]. The *crtE* gene has also been co-overexpressed with other genes to favor astaxanthin production with promising results. For example, the combined overexpression of genes *acaT*, *HMGS*, and *HMGR* from the mevalonate pathway led to 1.4-fold higher volumetric astaxanthin production [44]. Interestingly, when *crtE* overexpression was included in this combination, astaxanthin production increased by another 1.3-fold [44]. Our work focused solely on the overexpression of a single gene, as our goal was to evaluate the potential of the SREBP pathway regulation to promote carotenogenesis, which could be considered a limitation. However, our results open the opportunity for further optimization, such as combining *crtE* promoter replacement with the overexpression of other genes involved in carotenogenesis and/or the downregulation of competitive pathways.

Obtaining *X. dendrorhous* strains that overproduce carotenoids, even if they do not necessarily exhibit increased astaxanthin content, remains valuable for biotechnological purposes. Some studies have focused on increasing carotenoid production in *X. dendrorhous* mutants where astaxanthin production is blocked, leading to the accumulation of β-carotene instead [45]. One notable application of this is the production of zeaxanthin, an essential carotenoid for preventing macular degeneration, which *X. dendrorhous* does not naturally produce [46]. Zeaxanthin production in *X. dendrorhous* was achieved in a β-carotene accumulating strain by overexpressing endogenous genes *HMGR*, *crtE*, and *crtYB*, and expressing an optimized bacterial *crtZ* gene, encoding a beta-carotene hydroxylase. These genetic modifications resulted in a four-fold increase in total carotenoids, 68% of which were zeaxanthin [38]. Another example is the production of phytoene, the first carotenoid in carotenoid synthesis. Phytoene is colorless and exhibits UV absorption within the range of 260 to 320 nm [39] and supplementation of phytoene has a photoprotective effect; it accumulates in the skin and helps to prevent inflammation-induced redness caused by sun exposure, thereby minimizing UV radiation-induced erythema [47]. Phytoene accumulation in *X. dendrorhous* was achieved by overexpressing the *HMGR*, *crtE*, and *crtYB* genes*,* combined with the disruption of the *crtI* gene, which encodes the phytoene desaturase. These genetic modifications directed the metabolic flux towards phytoene, resulting in its accumulation [39].

To further enhance astaxanthin production in the strains obtained in this work, overexpression of genes involved in the final steps of carotenoid synthesis, such as *crtS* and *crtR*, could be considered as when the transcript levels of these genes are low, the intermediate carotenoid fraction increases and the astaxanthin fraction decreases [23, 37, 48]. Overexpression of *crtS* has increased astaxanthin accumulation from 68 to 96% compared to the wild-type strain, while reducing the fraction of intermediate carotenoids in *X. dendrorhous* after 96 h of cultivation [49]. In this context, the *X. dendrohous* gene *DAP1* was recently described, which plays a crucial role in the final steps of astaxanthin synthesis [40]. Deleting the *DAP1* gene resulted in a 30-fold reduction in astaxanthin and a 5.5-fold increment in β-carotene accumulation. Moreover, protein Dap1 coimmunoprecipitates with protein CrtS, suggesting a regulatory role of Dap1 on CrtS at the protein level [40]. Thus, overexpressing *DAP1* in the strains that were obtained in this work could further promote astaxanthin production. Taken together, these findings suggest that modifying the expression of genes such as *crtE*, *DAP1*, *crtR*, and/or *crtS*, in combination with genes from the mevalonate pathway like *HMGS* and/or *HMGR*, could promote carotenoid and astaxanthin production. This result could be more pronounced in strains with an activated SREBP pathway, as in strains CBS*.cyp61*^*−*^ and CBS*.SRE1N*.FLAG, especially when promoters of genes regulated by Sre1N are employed, as observed in this work.

## Conclusions

Replacing the native promoter of the *crtE* gene with that of the *HMGS* gene successfully enhanced carotenoid production in *Xanthophyllomyces dendrorhous* strains harboring the active Sre1N transcription factor. This strategy represents a novel approach to enhance the production of these biotechnological important compounds. Importantly, this genetic modification did not adversely affect growth or sterol production, suggesting that it did not significantly affect the physiology of this yeast and supporting its potential for improving carotenoid yields. While total carotenoid levels significantly increased, the rise in astaxanthin levels was less pronounced. Future research could consider complementary approaches, such as overexpressing downstream carotenogenic genes like *crtS* and *crtR*, involved in the astaxanthin production from β-carotene. Additionally, optimizing culture conditions may further enhance astaxanthin yield. Overall, this study presents a promising genetic engineering approach based on the SREBP pathway regulation to enhance carotenoid production, providing a valuable tool for biotechnological applications in the field of carotenoid production.

## Supplementary Information

Additional file 1.

## Data Availability

Not applicable.

## Abbreviations

SREBP: Sterol regulatory element-binding protein
MVA: Mevalonate
bHLH-LZ: Basic-helix-loop-helix leucine zipper
ER: Endoplasmic Reticulum
SCAP: SREBP cleavage-activating protein
S1P: Site-1 protease
S2P: Site-2 protease
SREs: Sterol Regulatory Elements
Insig: Insulin-induced gene
Sre1N: N-terminal domain of Sre1
OE-PCR: Overlap extension PCR

## References

[1] Higuera-Ciapara I, Félix-Valenzuela L, Goycoolea FM. Astaxanthin: a review of its chemistry and applications. Crit Rev Food Sci Nutr. 2006;46:185–96. 16431409 10.1080/10408690590957188

[2] Galasso C, Corinaldesi C, Sansone C. Carotenoids from marine organisms: biological functions and industrial applications. Antioxidants. 2017;6:96. 29168774 10.3390/antiox6040096 PMC5745506

[3] Johnson EA. *Phaffia rhodozyma*: colorful odyssey. Int Microbiol. 2003;6:169–74. 12898396 10.1007/s10123-003-0130-3

[4] Schmidt I, Schewe H, Gassel S, Jin C, Buckingham J, Hümbelin M, Sandmann G, Schrader J. Biotechnological production of astaxanthin with *Phaffia rhodozyma*/*Xanthophyllomyces dendrorhous*. Appl Microbiol Biotechnol. 2011;89:555–71. 21046372 10.1007/s00253-010-2976-6

[5] Rodriguez-Saiz M, de la Fuente JL, Barredo JL. *Xanthophyllomyces dendrorhous* for the industrial production of astaxanthin. Appl Microbiol Biotechnol. 2010;88:645–58. 20711573 10.1007/s00253-010-2814-x

[6] Mata-Gomez LC, Montanez JC, Mendez-Zavala A, Aguilar CN. Biotechnological production of carotenoids by yeasts: an overview. Microb Cell Fact. 2014;13:12. 24443802 10.1186/1475-2859-13-12 PMC3922794

[7] Torres-Haro A, Verdín J, Kirchmayr MR, Arellano-Plaza M. Metabolic engineering for high yield synthesis of astaxanthin in *Xanthophyllomyces dendrorhous*. Microb Cell Fact. 2021;20:175. 34488760 10.1186/s12934-021-01664-6 PMC8420053

[8] Breitenbach J, Visser H, Verdoes JC, van Ooyen AJ, Sandmann G. Engineering of geranylgeranyl pyrophosphate synthase levels and physiological conditions for enhanced carotenoid and astaxanthin synthesis in *Xanthophyllomyces dendrorhous*. Biotechnol Lett. 2010;33:755–61. 21165672 10.1007/s10529-010-0495-2

[9] Alcaíno J, Romero I, Niklitschek M, Sepúlveda D, Rojas MC, Baeza M, Cifuentes V. Functional characterization of the *Xanthophyllomyces dendrorhous* farnesyl pyrophosphate synthase and geranylgeranyl pyrophosphate synthase encoding genes that are involved in the synthesis of isoprenoid precursors. PLoS ONE. 2014;9: e96626. 24796858 10.1371/journal.pone.0096626 PMC4010515

[10] Bien C, Espenshade P. Sterol regulatory element binding proteins in fungi: hypoxic transcription factors linked to pathogenesis. Eukaryot Cell. 2010;9:352–9. 20118213 10.1128/EC.00358-09 PMC2837984

[11] Goldstein JL, DeBose-Boyd RA, Brown MS. Protein sensors for membrane sterols. Cell. 2006;124:35–46. 16413480 10.1016/j.cell.2005.12.022

[12] Moon YA, Liang G, Xie X, Frank-Kamenetsky M, Fitzgerald K, Koteliansky V, Brown MS, Goldstein JL, Horton JD. The Scap/SREBP pathway is essential for developing diabetic fatty liver and carbohydrate-induced hypertriglyceridemia in animals. Cell Metab. 2012;15:240–6. 22326225 10.1016/j.cmet.2011.12.017 PMC3662050

[13] Wu X, Yan R, Cao P, Qian H, Yan N. Structural advances in sterol-sensing domain-containing proteins. Trends Biochem Sci. 2022;47:289–300. 35012873 10.1016/j.tibs.2021.12.005

[14] Brown MS, Goldstein JL. A proteolytic pathway that controls the cholesterol content of membranes, cells, and blood. Proc Natl Acad Sci U S A. 1999;96:11041–8. 10500120 10.1073/pnas.96.20.11041 PMC34238

[15] Rawson RB. Control of lipid metabolism by regulated intramembrane proteolysis of sterol regulatory element binding proteins (SREBPs). Biochem Soc Symp. 2003;70:221–31. 10.1042/bss0700221 14587295

[16] Osborne TF, Espenshade PJ. Evolutionary conservation and adaptation in the mechanism that regulates SREBP action: what a long, strange tRIP it’s been. Genes Dev. 2009;23:2578–91. 19933148 10.1101/gad.1854309 PMC2779761

[17] Hughes AL, Todd BL, Espenshade PJ. SREBP pathway responds to sterols and functions as an oxygen sensor in fission yeast. Cell. 2005;120:831–42. 15797383 10.1016/j.cell.2005.01.012

[18] Chun CD, Liu OW, Madhani HD. A link between virulence and homeostatic responses to hypoxia during infection by the human fungal pathogen *Cryptococcus neoformans*. PLoS Pathog. 2007;3: e22. 17319742 10.1371/journal.ppat.0030022 PMC1803011

[19] Chang YC, Bien CM, Lee H, Espenshade PJ, Kwon-Chung KJ. Sre1p, a regulator of oxygen sensing and sterol homeostasis, is required for virulence in *Cryptococcus neoformans*. Mol Microbiol. 2007;64:614–29. 17462012 10.1111/j.1365-2958.2007.05676.x

[20] Gómez M, Campusano S, Gutiérrez MS, Sepúlveda D, Barahona S, Baeza M, Cifuentes V, Alcaíno J. Sterol regulatory element-binding protein Sre1 regulates carotenogenesis in the red yeast *Xanthophyllomyces dendrorhous*. J Lipid Res. 2020;61:1658–74. 32933952 10.1194/jlr.RA120000975 PMC7707178

[21] Gómez M, Baeza M, Cifuentes V, Alcaíno J. The SREBP (sterol regulatory element-binding protein) pathway: a regulatory bridge between carotenogenesis and sterol biosynthesis in the carotenogenic yeast *Xanthophyllomyces dendrorhous*. Biol Res. 2021;54:34. 34702374 10.1186/s40659-021-00359-x PMC8549280

[22] Gutiérrez MS, Campusano S, González AM, Gómez M, Barahona S, Sepúlveda D, Espenshade PJ, Fernández-Lobato M, Baeza M, Cifuentes V, Alcaíno J. Sterol regulatory element-binding protein (Sre1) promotes the synthesis of carotenoids and sterols in *Xanthophyllomyces dendrorhous*. Front Microbiol. 2019;10:586. 30984134 10.3389/fmicb.2019.00586 PMC6449425

[23] Gómez M, Gutiérrez MS, González AM, Gárate-Castro C, Sepúlveda D, Barahona S, Baeza M, Cifuentes V, Alcaíno J. Metallopeptidase Stp1 activates the transcription factor Sre1 in the carotenogenic yeast *Xanthophyllomyces dendrorhous*. J Lipid Res. 2020;61:229–43. 31806730 10.1194/jlr.RA119000431 PMC6997601

[24] Loto I, Gutiérrez MS, Barahona S, Sepúlveda D, Martínez-Moya P, Baeza M, Cifuentes V, Alcaíno J. Enhancement of carotenoid production by disrupting the C22-sterol desaturase gene (*CYP61*) in *Xanthophyllomyces dendrorhous*. BMC Microbiol. 2012;12:235. 23075035 10.1186/1471-2180-12-235 PMC3552872

[25] Adrio JL, Veiga M. Transformation of the astaxanthin-producing yeast *Phaffia rhodozyma*. Biotechnol Tech. 1995;9:509–12. 10.1007/BF00311214 7586031

[26] Kim IG, Nam SK, Sohn JH, Rhee SK, An GH, Lee SH, Choi ES. Cloning of the ribosomal protein L41 gene of *Phaffia rhodozyma* and its use a drug resistance marker for transformation. Appl Environ Microbiol. 1998;64:1947–9. 9572978 10.1128/aem.64.5.1947-1949.1998 PMC106257

[27] Livak KJ, Schmittgen TD. Analysis of relative gene expression data using real-time quantitative PCR and the 2-∆∆*C*T method. Methods. 2001;25:402–8. 11846609 10.1006/meth.2001.1262

[28] Boyle JS, Lew AM. An inexpensive alternative to glassmilk for DNA purification. Trends Genet. 1995;11:8. 7900196 10.1016/s0168-9525(00)88977-5

[29] Sambrook J, Russell DW. Molecular cloning: a laboratory manual. 3rd ed. Cold Spring Harbor: Cold Spring Harbor Laboratory Press; 2001.

[30] Chomczynski P, Sacchi N. Single-step method of RNA isolation by acid guanidinium thiocyanate-phenol-chloroform extraction. Anal Biochem. 1987;162:156–9. 2440339 10.1006/abio.1987.9999

[31] An G-H, Schuman DB, Johnson EA. Isolation of *Phaffia rhodozyma* mutants with increased astaxanthin content. Appl Environ Microbiol. 1989;55:116–24. 16347815 10.1128/aem.55.1.116-124.1989 PMC184064

[32] Mercadante AZ, Egeland ES. Carotenoids handbook. Basel, Boston, Berlin: Birkhäuser Verlag; 2004.

[33] Gassel S, Schewe H, Schmidt I, Schrader J, Sandmann G. Multiple improvement of astaxanthin biosynthesis in *Xanthophyllomyces dendrorhous* by a combination of conventional mutagenesis and metabolic pathway engineering. Biotechnol Lett. 2013;35:565–9. 23187756 10.1007/s10529-012-1103-4

[34] Li Z, Yang H, Zheng C, Du X, Ni H, He N, Yang L, You L, Zhu Y, Li L. Effectively improve the astaxanthin production by combined additives regulating different metabolic nodes in *Phaffia rhodozyma*. Front Bioeng Biotechnol. 2021;9:812309. 35111739 10.3389/fbioe.2021.812309 PMC8801872

[35] Gervasi T, Santini A, Daliu P, Salem AZM, Gervasi C, Pellizzeri V, Barrega L, De Pasquale P, Dugo G, Cicero N. Astaxanthin production by *Xanthophyllomyces dendrorhous* growing on a low cost substrate. Agroforest Syst. 2020;94:1229–34.

[36] Lai JX, Liu WP, Bu J, Chen X, Hu BB, Zhu MJ. Enhancement of astaxanthin production from food waste by Phaffia rhodozyma screened by flow cytometry and feed application potential. Biotechnol Appl Biochem. 2023;70:1817–29. 37278155 10.1002/bab.2484

[37] Hara KY, Morita T, Endo Y, Mochizuki M, Araki M, Kondo A. Evaluation and screening of efficient promoters to improve astaxanthin production in *Xanthophyllomyces dendrorhous*. Appl Microbiol Biotechnol. 2014;98:6787–93. 24737060 10.1007/s00253-014-5727-2

[38] Pollmann H, Breitenbach J, Sandmann G. Engineering of the carotenoid pathway in *Xanthophyllomyces dendrorhous* leading to the synthesis of zeaxanthin. Appl Microbiol Biotechnol. 2016;101:103–11. 27527661 10.1007/s00253-016-7769-0

[39] Pollmann H, Breitenbach J, Sandmann G. Development of *Xanthophyllomyces dendrorhous* as a production system for the colorless carotene phytoene. J Biotechnol. 2017;247:34–41. 28263769 10.1016/j.jbiotec.2017.02.027

[40] González AM, Venegas M, Barahona S, Gómez M, Gutiérrez MS, Sepúlveda D, Baeza M, Cifuentes V, Alcaíno J. Damage response protein 1 (Dap1) functions in the synthesis of carotenoids and sterols in *Xanthophyllomyces dendrorhous*. J Lipid Res. 2022;63:100175. 35120994 10.1016/j.jlr.2022.100175 PMC8953664

[41] Yamamoto K, Hara KY, Morita T, Nishimura A, Sasaki D, Ishii J, Ogino C, Kizaki N, Kondo A. Enhancement of astaxanthin production in *Xanthophyllomyces dendrorhous* by efficient method for the complete deletion of genes. Microb Cell Fact. 2016;15:155. 27624332 10.1186/s12934-016-0556-x PMC5022159

[42] Venegas M, Barahona S, González AM, Sepúlveda D, Zúñiga GE, Baeza M, Cifuentes V, Alcaíno J. Phenotypic analysis of mutants of ergosterol biosynthesis genes (*ERG3* and *ERG4*) in the red yeast *Xanthophyllomyces dendrorhous*. Front Microbiol. 2020;11:1312. 32612595 10.3389/fmicb.2020.01312 PMC7309136

[43] Horton JD, Shah NA, Warrington JA, Anderson NN, Park SW, Brown MS, Goldstein JL. Combined analysis of oligonucleotide microarray data from transgenic and knockout mice identifies direct SREBP target genes. Proc Natl Acad Sci U S A. 2003;100:12027–32. 14512514 10.1073/pnas.1534923100 PMC218707

[44] Hara KY, Morita T, Mochizuki M, Yamamoto K, Ogino C, Araki M, Kondo A. Development of a multi-gene expression system in *Xanthophyllomyces dendrorhous*. Microb Cell Fact. 2014;13:175. 25471659 10.1186/s12934-014-0175-3 PMC4264253

[45] Girard P, Falconnier B, Bricout J, Vladescu B. β-Carotene producing mutants of *Phaffia rhodozyma*. Appl Microbiol Biotechnol. 1994;41:183–91.

[46] Andrewes AG, Phaff HJ, Starr MP. Carotenoids of *Phaffia rhodozyma*, a red-pigmented fermenting yeast. Phytochemistry. 1976;15:1003–7.

[47] von Oppen-Bezalel L, Fishbein D, Havas F, Ben-Chitrit O, Khaiat A. The photoprotective effects of a food supplement tomato powder rich in phytoene and phytofluene, the colorless carotenoids, a preliminary study. Glob Dermatol. 2015. 10.15761/GOD.1000149.

[48] Wan X, Zhou XR, Moncalian G, Su L, Chen WC, Zhu HZ, Chen D, Gong YM, Huang FH, Deng QC. Reprogramming microorganisms for the biosynthesis of astaxanthin via metabolic engineering. Prog Lipid Res. 2021;81:101083. 33373616 10.1016/j.plipres.2020.101083

[49] Contreras G, Barahona S, Rojas MC, Baeza M, Cifuentes V, Alcaíno J. Increase in the astaxanthin synthase gene (*crtS*) dose by in vivo DNA fragment assembly in *Xanthophyllomyces dendrorhous*. BMC Biotechnol. 2013;13:84. 24103677 10.1186/1472-6750-13-84 PMC3852557

[50] Zwietering MH, Jongenburger I, Rombouts FM. Van’t Riet K: modeling of the bacterial growth curve. Appl Environ Microbiol. 1990;56:1875–81. 16348228 10.1128/aem.56.6.1875-1881.1990 PMC184525

[51] Niklitschek M, Alcaíno J, Barahona S, Sepúlveda D, Lozano C, Carmona M, Marcoleta A, Martínez C, Lodato P, Baeza M, Cifuentes V. Genomic organization of the structural genes controlling the astaxanthin biosynthesis pathway of *Xanthophyllomyces dendrorhous*. Biol Res. 2008;41:93–108. 18769767 10.4067/S0716-97602008000100011

