# Supplementary material for: Increasing carotenoid production in Xanthophyllomyces dendrorhous/Phaffia rhodozyma: SREBP pathway activation and promoter engineering
Source: Biol Res. 2024 Nov 5;57:78. doi: 10.1186/s40659-024-00559-1 (PMC11536662; doi:10.1186/s40659-024-00559-1)
Supplement: Supplementary file 1 — Additional file 1. [file 40659_2024_559_MOESM1_ESM.docx]

Supplementary Material

# Supplementary Figures and Tables

## Supplementary Figure


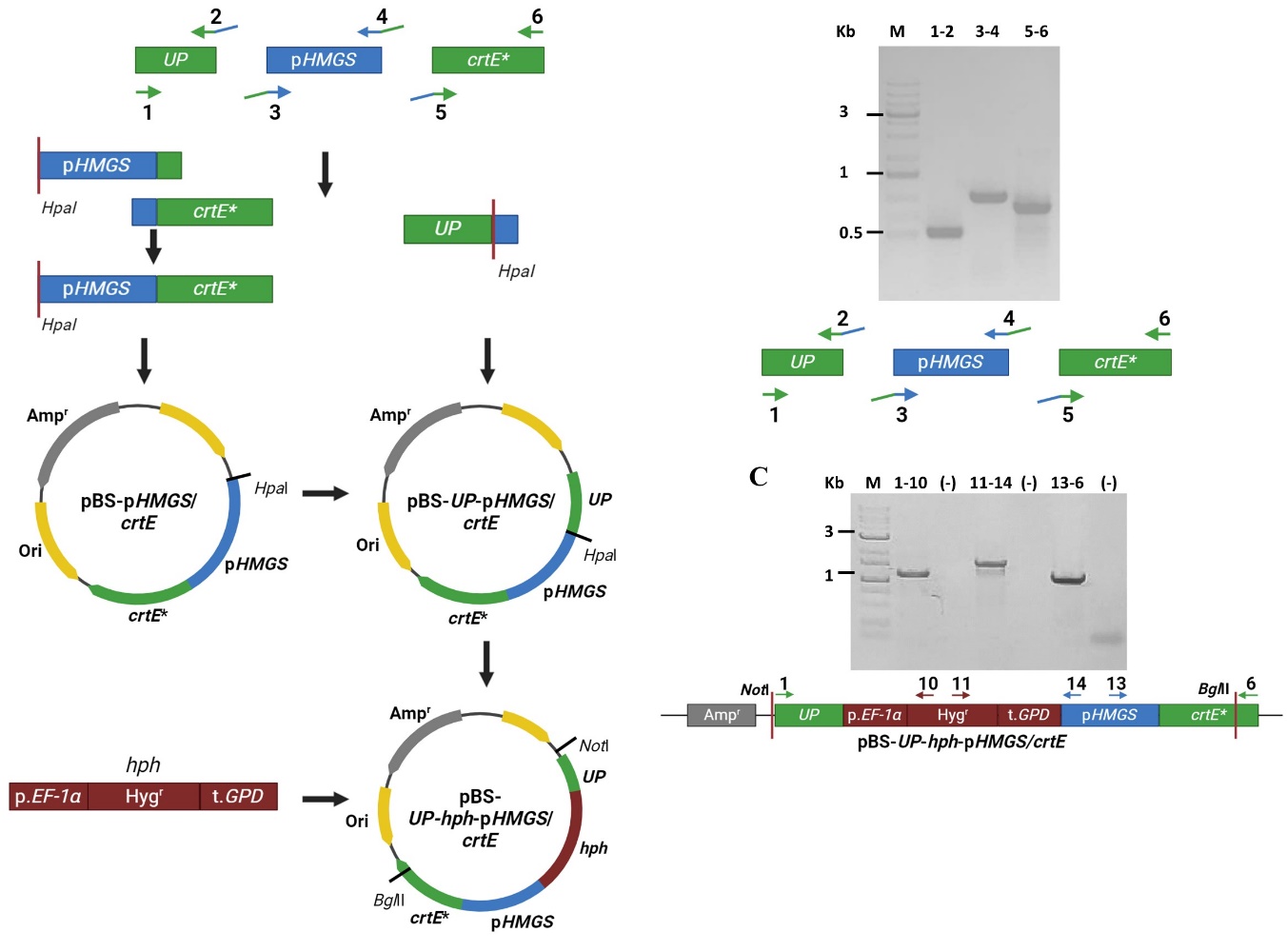


**Supplementary Figure 1.** **Representation, construction, and analysis of the pBS-*Up-hph-pHMGS*/*crtE* vector. A)** Fragments of interest were amplified, including an upstream region of the *crtE* gene promoter (*UP*), the *HMGS* gene promoter region (p*HMGS*), and a portion from exon 1 to 81 bp of exon 4 of the *crtE* gene (*crtE**). Specific primers were designed (Table 1.2.1 in the supplementary material) to generate amplicons with 10 complement bases, allowing the fusion of p*HMGS* and *crtE** through *Overlap Extension* PCR (OE-PCR). Subsequently, the generated fragment was inserted at the *Eco*RV site of plasmid pBS, giving rise to the pBS-*pHMGS*/*crtE* vector with the *Hpa*I restriction site added upstream of p*HMGS*. The UP region, also amplified by PCR, was inserted at the *Hpa*I site of pBS-*pHMGS*/*crtE*, resulting in the pBS-*UP-pHMGS*/*crtE** vector. The UP region also included a *Hpa*I restriction site to insert at this site the Hygromycin B resistance module (hph) in pBS-*UP-pHMGS*/*crtE**, obtaining the vector pBS-*Up-hph-pHMGS*/*crtE**. The figure also shows the enzymatic restriction sites, *Not*I in the *UP* region, and *Bgl*II in the exon 4 region, enzymes that were used to release the DNA fragment used to transform the strains of this study: wild-type strain CBS*6938* and the mutant strains CBS.*cyp61^-^* and CBS.*SRE1N.FLAG* of *X. dendrorhous*. **B)** The amplification of each DNA fragment was confirmed by agarose gel electrophoresis. The numbers associated with each lane represent the primers used in the PCR reactions, and DNA from the wild-type *X. dendrorhous* strain CBS*6938* was used as template. **C)** Verification of the pBS-*Up-hph-pHMGS*/*crtE** vector was performed by amplifying three different regions of the plasmid with specific primers (Table 1.2.1 in the supplementary material). In the agarose gel image, the numbers at the top of the lanes indicate the primers used in the PCR reactions. M = GeneRuler^TM^ 1 kb Plus DNA Ladder molecular weight marker. (-) represents a control without DNA.

## Supplementary Table

### Supplementary Table 1. Primers used in this work.

| **N°** | **Name** | **Sequence (5´- 3´)** | **Orientation** |
| --- | --- | --- | --- |
| 1 | FwUp** | GTTGGACGAATGTAGCCG | D |
| 2 | RvUp* | GTTCGAACTGG**GTTAAC**CATCAAGGAGAGTACTCTCG | R |
| 3 | Fhmgs* | CTCCTTGATG**GTTAAC**CCAGTTCGAACGAGAGTATCGT | D |
| 4 | RvHmgs | TCGCGTAATCCATAACTGGATATCTAGATTGAGAT | R |
| 5 | Fwcrte | ATATCCAGTTATGGATTACGCGAACATCCTC | F |
| 6 | Rvcrte** | GGAGGAATTACAGGCATGGGT | R |
| 7 | Gpd.F | ACGGTTCTCTCCAAACCCTC | D |
| 8 | H.out.R | TCCATCACAGTTTGCCAGTG | R |
| 9 | P.tef.F | GGCTCATCAGCCGACAGTTCA | D |
| 10 | Hyg.Rev.In** | TGTCAAGCACTTCCGGAATCG | R |
| 11 | Int.Hyg.F** | GTGTCACGTTGCAAGACCTG | D |
| 12 | FwUp2** | GAATTCCAACACAAGCTGGA | D |
| 13 | FwHmgs2** | GATTGACCTTGAGTACGCGG | D |
| 14 | RvHmgs2** | CGGCCAAGGTATGTAAGTTC | R |
| 15 | Rvcrte2 | CCAACAACGTTCTGGATGAC | R |
| 16 | M13Fw | GTAAAACGACGGCCAGT | D |
| 17 | M13Rv | GTCATAGCTGTTTCCTG | R |
| 18 | FwD2check | AGACCGAGGAGAAGGTTTCATG | D |
| 19 | FUP2 | CTTAAAAGCTCGCACTGGTC | D |
| 20 | crtE.out.F1 | ATGACTCTTGCCGGCGATTTGA | D |
| 21 | R1.crtE | AAGCTGGACAAAGTCTCTAC | R |
| 22 | FwUp.Sc | GTTGGACGAATGTAGCCG | D |
| 23 | RvHyg.Sc | GTCGGCTGATGAGCCGAT | R |
| 24 | FwHMGS.Sc | CCATCATCTCCGTCATCT | D |
| 25 | RvHMGS.Sc | CCGACTCAGTAGAGTTCT | R |
| 26 | FwHMGS2.Sc | CAGAGTACACCGTATCGA | D |
| 27 | RvcrtE.Sc | TACGGTTCAAGGAGCACG | R |
| 28 | FwcrtE.Sc | CGTGCTCCTTGAACCGTATC | D |
| 29 | FwcrtE2.Sc | CGCTAGCTTATTGTGAGTC | D |
| 30 | RvcrtE2.Sc | AGACTCACAATAAGCTAGCG | R |
| 31 | RvcrtE3.Sc | CTCTTGATAAGCCAGAAAGT | R |
| 32 | mActF-RT | CCGCCCTCGTGATTGATAAC | F |
| 33 | mActR-RT | TCACCAACGTAGGAGTCCTT | R |
| 34 | mcrtEF-RT | TGTTGGCATGCTACATACCCG | F |
| 35 | mcrtER-RT | GTTGGGCGAAGCTTGAAGAT | R |
| 36 | hmgsRT2-F | AAGCAGGTTGAGCCTGGAATGA | F |
| 37 | HmgrsRT2-R | AAAGCGGTTGAGCTCTTGACCT | R |
| Primers D = Forward; R = Reverse.* Primers with an added *Hpa*I sequence, highlighted in bold. ** Intermediate primers of the construction module. | | | |
